# Supplementary material for: The global extent of the grassland biome and implications for the terrestrial carbon sink
Source: Nat Ecol Evol. 2026 Jan 27;10(2):246–57. doi: 10.1038/s41559-025-02955-6 (PMC12929054; doi:10.1038/s41559-025-02955-6)
Supplement: Supplementary file 1 — Additional Methods, Supplementary Figs. 1–5 and Tables 1–6. [file 41559_2025_2955_MOESM1_ESM.pdf]

nature ecology & evolution

Supplementary information https://doi.org/10.1038/s41559-025-02955-6

The global extent of the grassland biome and implications for the terrestrial carbon sink

In the format provided by the authors and unedited
